# Bleeding in Antiplatelet Therapy: A Narrative Review of Clinical Evidence and the Roles of Pharmacists and Nurses

**DOI:** 10.31083/RCM45077

**Published:** 2025-11-25

**Authors:** Feng Xu, Zhi-Hui Zhang, Liu-Cheng Li, Kai-Li Mao, Zi-Ying Huang

**Affiliations:** ^1^Department of Pharmacy, Ningbo No.2 Hospital, 315010 Ningbo, Zhejiang, China; ^2^Institute of Vascular Anomalies, Shanghai TCM-Integrated Hospital, Shanghai University of Traditional Chinese Medicine, 200082 Shanghai, China; ^3^Department of Pharmacy, Sir Run Run Shaw Hospital, Zhejiang University School of Medicine, 310016 Hangzhou, Zhejiang, China; ^4^Department of Pharmacy, The Quzhou Affiliated Hospital of Wenzhou Medical University, Quzhou People’s Hospital, 324000 Quzhou, Zhejiang, China; ^5^Department of Pharmacy, Danzhou People’s Hospital, 571700 Danzhou, Hainan, China

**Keywords:** antiplatelet, bleeding, pharmacists, nurses, patient management

## Abstract

To accumulate and evaluate current evidence on bleeding complications associated with antiplatelet therapy and the specific contributions of pharmacists and nurses to bleeding-risk mitigation. Antiplatelet agents prevent arterial thrombosis by inhibiting platelet aggregation through blocking cyclooxygenase-1, P2Y_12_ receptors, glycoprotein (GP) IIb/IIIa receptors, or phosphodiesterase pathways. These mechanisms simultaneously impair primary hemostasis, increasing the risk of intracranial, gastrointestinal, or other clinically significant bleeding. Bleeding risk is dose-, duration-, and drug-dependent; meanwhile, dual antiplatelet therapy (DAPT) and concurrent use of anticoagulants, non-steroidal anti-inflammatory drugs (NSAIDs), corticosteroids, or proton pump inhibitors all amplify the risk. Patient-specific factors, likely older ages, anemia, renal or hepatic impairment, prior bleeding, cancer, diabetes, and frailty further increase the hazard. Shortened DAPT or P2Y_12_ inhibitor monotherapy reduces bleeding without increasing thrombotic events. Pharmacists optimize regimens, screen for interactions, educate patients, and co-develop institutional protocols; nurses monitor early signs of bleeding, ensure adherence, and coordinate multidisciplinary care. Both roles demonstrably decrease the incidence and severity of bleeding. Individualized antiplatelet strategies, guided by refined risk-stratification tools and delivered through pharmacist-nurse integrated care models, can maximize antithrombotic benefit while minimizing bleeding harm. Thus, large prospective trials and cost-effectiveness analyses are warranted to validate these multidisciplinary interventions.

## 1. Introduction

Antiplatelet agents are fundamental in cardiovascular therapy, providing substantial protection against arterial thrombosis [1]. Their mechanism, which primarily involves the inhibition of platelet aggregation, is key to their effectiveness in preventing thrombotic events across various cardiovascular conditions [2, 3]. However, this therapeutic benefit is offset by an inherent risk of bleeding complications [4], ranging from minor, self-limiting bruising to severe hemorrhagic events, such as intracranial or gastrointestinal bleeding, which can be fatal [5, 6]. The incidence and severity of these bleeding events are influenced by a complex interplay of factors, including the specific antiplatelet agent used, its dosage and duration of treatment, and patient-specific variables such as age, genetic polymorphisms affecting drug metabolism, comorbidities like renal or hepatic impairment, and concurrent medication use [7, 8]. Antiplatelet therapy is employed in various clinical contexts [9]. The highest bleeding risk is observed in (i) patients with atrial fibrillation undergoing percutaneous coronary intervention (PCI) and receiving triple antithrombotic therapy (oral anticoagulant plus dual antiplatelet therapy); (ii) post-PCI patients on dual antiplatelet therapy (DAPT); and (iii) patients with atherosclerotic cardiovascular disease (ASCVD) treated with dual antithrombotic therapy, combining low-dose rivaroxaban with antiplatelet agents [9]. In these scenarios, the cumulative anti-hemostatic effects significantly heighten the risk of major or fatal bleeding, requiring meticulous risk stratification and mitigation.

The clinical challenge of managing bleeding events extends beyond their immediate treatment, necessitating a nuanced approach to risk stratification and mitigation—areas where current clinical evidence remains insufficient. Guidelines for managing hemorrhage in antiplatelet-treated patients are often based on expert consensus rather than robust trial data. In this context, the integration of multidisciplinary care becomes essential. Pharmacists and nurses, as key members of the healthcare team, play critical roles in optimizing patient outcomes [10, 11, 12]. Pharmacists apply their expertise in pharmacotherapy to refine medication regimens, monitor drug interactions, and implement protocols to reduce hemorrhage risk. Nurses contribute through vigilant monitoring, early detection of bleeding signs, and patient-centered education, which promotes adherence and safety. Together, their efforts create a comprehensive framework for assessing and mitigating bleeding risk.

This review synthesizes existing evidence on bleeding complications associated with antiplatelet agents and examines the vital contributions of pharmacists and nurses in hospital settings. By highlighting their roles, this review aims to enhance understanding of hemorrhage risk management and improve the quality of care for patients receiving antiplatelet therapy.

## 2. Literature Review

This study conducted a literature review to assess updated research on bleeding risks associated with antiplatelet therapy. Data were gathered from widely used databases, including PubMed, Web of Science, and China National Knowledge Infrastructure, with a search cutoff date of June 2025. The review incorporated observational studies, randomized controlled trials, meta-analyses, and guideline consensus documents. Search keywords included antiplatelet, aspirin, indobufen, ozagrel, clopidogrel, ticagrelor, prasugrel hydrochloride, cangrelor, tirofiban, abciximab, eptifibatide, cilostazol, batifiban, dipyridamole, anagrelide, beraprost, ticlopidine, sarpogrelate, vorapaxar, bleeding, pharmacist, and nurse.

## 3. Indications of Antiplatelet Drugs and Influencing Factors of Bleeding

**Fig. 1.**
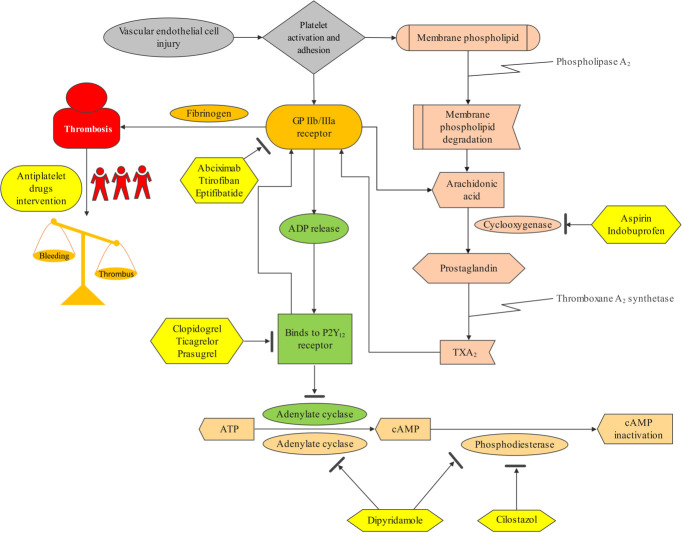
**Mechanism of antiplatelet drugs in preventing and treating thrombosis**. Abbreviations: ADP, adenosine diphosphate; ATP, adenosine triphosphate; cAMP, cyclic adenosine-3^′^,5^′^-monophosphate; GP, glycoprotein; TXA_2_, thromboxane A2.

**Fig. 2.**
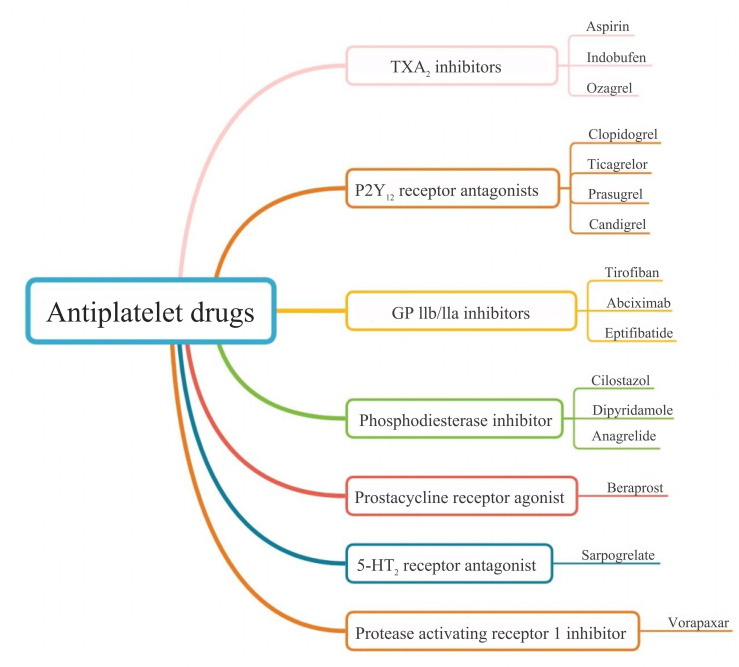
**Emerging classification of antiplatelet drugs**. 5-HT2, 5-hydroxytryptamine receptor 2.

Platelets play a central role in arterial thrombosis formation, and antiplatelet therapy is integral to treating cardiovascular diseases and preventing strokes (Fig. 1) [13, 14, 15]. The first antiplatelet drug, aspirin (acetylsalicylic acid), was first synthesized and used in 1899. Since then, numerous antiplatelet agents with varying mechanisms of action have been developed, including thromboxane A_2_ inhibitors, P2Y_12_ receptor antagonists, glycoprotein (GP) IIb/IIIa antagonists, and phosphodiesterase inhibitors (Fig. 2). Antiplatelet therapy is commonly indicated for PCI, with decisions regarding DAPT and its duration depending on the clinical diagnosis and the status of interventional therapy [16]. The clinical goal is to balance hemorrhage reduction and mortality while optimizing antiplatelet efficacy. While antiplatelet therapy is crucial for patients with ASCVD, bleeding risk must also be carefully considered, particularly when combined with DAPT or anticoagulant drugs [16, 17].

The commonly used classification systems at present include: The Bleeding Academic Research Consortium (BARC) classification divides bleeding into types 0 to 5, among which type 3 and above are major bleeding that requires clinical attention [18]. The thrombolysis in myocardial infarction (TIMI) classification is divided into major, minor and minimal bleeding and is widely used in cardiovascular clinical trials [19]. Bleeding complications encompass intracranial hemorrhage, gastrointestinal bleeding, hematuria, and epistaxis. Life-threatening bleeding refers to significant hemorrhage that results in hypovolemic shock or severe hypotension, potentially accompanied by a decrease in hemoglobin exceeding 50 g/L, requiring the transfusion of ≥5 units of red blood cells, and possibly leading to death [20]. Commonly used antiplatelet agents include aspirin and P2Y_12_ inhibitors (clopidogrel and ticagrelor) [21]. Aspirin inhibits cyclooxygenase-1, blocking prostaglandin synthesis, which weakens the gastric mucosal barrier, leading to gastric mucosal damage. A two-by-two factorial trial involving 6100 patients with mild ischemic stroke or high-risk transient ischemic attacks found that 0.4% of patients treated with aspirin experienced moderate to severe bleeding [22]. Another study identified a significant association between positive fecal occult blood in patients treated with antiplatelet therapy (aspirin or clopidogrel) and the degree of frailty in the elderly. The risk of positive fecal occult blood increased progressively with frailty, with the average risk increasing by 1.36 times for each degree of frailty [23]. For patients on aspirin, timely assessment and management of geriatric frailty and bleeding risk are crucial for ensuring safe antithrombotic therapy, especially in those receiving DAPT [23, 24, 25]. Clopidogrel, a P2Y_12_ receptor inhibitor, irreversibly blocks platelet function within 7 to 10 days, while ticagrelor, a reversible P2Y_12_ receptor inhibitor, impairs platelet function for 3 to 5 days. While clopidogrel does not directly damage the gastric mucosa, it may delay the healing of gastric mucosal lesions, increasing the risk of gastrointestinal bleeding.

A recent study involving 383 patients aged ≥65 with coronary heart disease (CHD) treated with ticagrelor for 1 year post-discharge, followed by 1 year of follow-up, identified several factors influencing ticagrelor-induced bleeding [26]. Univariate analysis indicated that lower hemoglobin levels, type 2 diabetes, and proton pump inhibitor (PPI) use were risk factors for bleeding, while the use of beta blockers, calcium channel blockers, and nitrates appeared protective [26]. Multivariate analysis confirmed that decreased hemoglobin, type 2 diabetes, and PPI use were significant risk factors for bleeding, with beta blocker use being the only protective factor. The bleeding mechanism was linked to the reversible inhibition of the adenosine diphosphate receptor, leading to inhibited platelet aggregation and activity [26]. PPI use was positively associated with bleeding events, suggesting that it serves as a marker for a higher incidence of bleeding rather than a direct cause. Lower hemoglobin levels were inversely associated with ticagrelor use, with anemia in elderly CHD patients indicating an elevated bleeding risk. Type 2 diabetes was positively correlated with bleeding events, while beta blockers were negatively associated with bleeding in ticagrelor users, possibly due to their effect of reducing intravascular blood flow and promoting clotting [26]. These findings imply that beta blockers may help reduce the risk of ticagrelor-induced bleeding. Consequently, elderly patients on ticagrelor should focus on managing blood sugar, correcting anemia, and addressing gastrointestinal conditions to mitigate bleeding risk. The 2023 Canadian Cardiovascular Society (CCS)/Canadian Association of Interventional Cardiology (CAIC) Guidelines outline bleeding risk factors for antiplatelet therapy, including low platelet count, anemia, renal or liver disease, active cancer, age ≥75, stroke, intracranial hemorrhage, brain arteriovenous malformation, bleeding diathesis, prior bleeding or transfusion, planned surgery on DAPT, recent trauma or surgery, non-steroidal anti-inflammatory drugs (NSAIDs), steroids, and oral anticoagulants [26].

Periprocedural intravenous antiplatelet therapy is increasingly used during acute carotid stenting with mechanical thrombectomy. A multicentre cohort study compared low-dose intravenous cangrelor with GP IIb/IIIa inhibitors (tirofiban/eptifibatide) in patients with acute tandem lesions [27]. Symptomatic intracranial hemorrhage occurred in 3.3% of cangrelor-treated patients versus 12.1% with GP IIb/IIIa inhibitors, while rates of parenchymal hematoma (17.9% vs 12.1%) and hemorrhagic infarction (21.4% vs 42.4%) were similar between groups [27]. Cangrelor’s ultra-short half-life (3–6 min) allows rapid reversal within 1 hour, obviating the need for platelet transfusion. These findings suggest comparable safety profiles, with cangrelor offering the additional advantage of immediate and reversible platelet inhibition.

## 4. Evidence-Based Comparison of Antiplatelet Regimens and Bleeding Risk

Emerging large clinical studies have compared the effects of different antiplatelet regimens on bleeding events and clinical outcomes across various disease states. The specific combination and duration of DAPT should be tailored to the severity of acute cardiocerebrovascular events, myocardial ischemia, and the patient’s bleeding risk profile [21, 24]. To prevent recurrent stroke, the American Heart Association/American Stroke Association (AHA/ASA) guidelines of 2021 recommend DAPT for 3 months following a transient ischemic attack, after which a single antiplatelet agent should be used [28]. Long-term antiplatelet therapy can reduce the frequency of ischemic cardiovascular and cerebrovascular events but may also increase the incidence of cerebral hemorrhage, particularly in patients with low thrombotic event risk, such as healthy individuals under 50 years of age [21]. A quantitative system for assessing thrombosis and bleeding risk will be necessary in the future. Compared to aspirin monotherapy, clopidogrel monotherapy significantly reduces the risk of type 3 and higher bleeding events (as defined by the Bleeding Academic Research Consortium), all-cause mortality, and stroke during the maintenance period following drug-eluting stent implantation [29].

In patients requiring antiplatelet monotherapy post-PCI, clopidogrel monotherapy has shown superiority over aspirin monotherapy in preventing future adverse events [29]. The combination of aspirin and P2Y_12_ inhibitors remains the standard antiplatelet regimen and is recommended to prevent ischemic complications immediately after PCI [30]. Thrombotic complications are most common in the first few months post-PCI, while the risk of bleeding stabilizes over time, supporting the concept of an “antiplatelet degradation ladder”, where discontinuation or dose reduction of antiplatelet agents may be considered [30]. A multi-center clinical trial published in JAMA involving 3045 PCI patients demonstrated that clopidogrel monotherapy following 1 month of combination therapy (aspirin and clopidogrel) significantly reduced the incidence of bleeding and cardiovascular events compared to 12 months of aspirin and clopidogrel [31]. Antiplatelet therapy is central to managing atherosclerotic and thrombotic diseases. In patients treated with PCI and other antiplatelet agents, strategies to minimize bleeding risk before, during, and after PCI are crucial. However, due to concerns about thrombosis and recurrent ischemia, extended DAPT durations are often employed [32]. Recent guidelines generally recommend a 6-month DAPT duration for patients with chronic coronary syndrome and 12 months for those with acute coronary syndrome (ACS) [32]. For patients at high bleeding risk, a shorter DAPT duration may be considered, ranging from 1 to 3 months after PCI in chronic coronary syndrome and 3 to 6 months in ACS patients [32].

In carotid stenting, DAPT offers advantages over monotherapy in reducing the risk of transient ischemic attacks but is associated with an increased risk of bleeding complications in patients undergoing carotid endarterectomy [33]. A network meta-analysis revealed no significant differences in major cardiac adverse events, clinical net adverse events, cardiac death, all-cause mortality, ischemic stroke, stent thrombosis, total bleeding, or major bleeding in patients with coronary artery disease receiving chronic maintenance antithrombotic therapy (aspirin, clopidogrel, ticagrelor, continuous DAPT, and aspirin plus low-dose anticoagulants) [34]. However, the incidence of ischemic stroke was lower with aspirin plus low-dose rivaroxaban compared to aspirin alone, while prolonged DAPT resulted in a higher total bleeding rate [34]. A recent two-by-two factorial trial found that 0.9% of patients with high-risk transient ischemic attacks or mild ischemic strokes treated with clopidogrel and aspirin experienced moderate to severe bleeding, a higher incidence than with aspirin alone, though this combination was associated with a lower occurrence of new strokes [22]. A network meta-analysis of 64 randomized controlled trials involving 102,735 patients showed that the major adverse cardiovascular event (MACE) rate for durable and biodegradable polymer stents was similar to that of bioabsorbable stents, regardless of DAPT duration [35]. When DAPT exceeded 12 months, fewer myocardial infarctions were observed in users of everolimus-eluting or zotamolimus-eluting stents, while the stent thrombosis rate for bioabsorbable stents was higher than that for the everolimus- or zotamolimus-eluting groups, irrespective of DAPT duration [35]. In a meta-analysis of 36,881 carotid endarterectomy and 150 carotid stenting procedures, no significant difference in stroke, transient ischemic attack, or death was found between single therapy and DAPT in carotid endarterectomy. However, dual therapy was associated with a higher risk of major bleeding, neck hematoma, and myocardial infarction [33]. No significant differences in major bleeding or hematoma formation were observed in carotid stenting, although DAPT reduced the incidence of transient ischemic attacks [33]. Individualized assessment, including demographic factors, angiographic features, platelet function testing, and rapid genotyping, has become a key approach to selecting the optimal antiplatelet therapy for minimizing severe and life-threatening bleeding [36]. The European Society of Cardiology (ESC) 2021 guidelines elevated aspirin monotherapy after transcatheter aortic valve replacement (TAVR) to a Class I (Grade A) recommendation because it significantly reduced major or life-threatening bleeding within 30 days compared with DAPT without any difference in ischemic events [37]. For patients with combined PCI or atrial fibrillation, short-term DAPT+oral anticoagulant (OAC) or OAC+clopidogrel dual regimens are adopted, and individualized selection should be made in combination with tools such as PREDICT-TAVR scoring to maximize the balance of thrombosis and bleeding risks [37].

The processes of absorption, metabolism, and excretion of antiplatelet drugs in patients with hepatic and renal insufficiency may lead to an increased risk of bleeding due to reduced clearance and prolonged half-life. Liver disease is a bleeding risk factor for antiplatelet decision-making [38]. Antiplatelet drugs such as abciximab, tirofiban and cilostazol that are not metabolized by the liver can be selected for patients with liver dysfunction, while clopidogrel that require liver metabolism should not be used in patients with severe liver insufficiency [39]. Renal disease is also a bleeding risk factor for antiplatelet [38, 40]. Abciximab, ticagrelor, and sargrexil may be used in patients with mild to moderate renal insufficiency, and the dose of GP IIb/IIIa receptor antagonists should be adjusted, while antiplatelet agents should be used with caution and the risk of thrombosis and bleeding should be weighed in patients with severe renal insufficiency and dialysis [39]. Studies of the use of antiplatelet drugs in patients with end-stage renal disease and/or dialysis are often underrepresented or outside of clinical trial inclusion criteria, leaving significant room for future researches.

Based on the recent systematic review, low-dose aspirin (75–100 mg) remains the best-evidenced antiplatelet agent in pregnancy and can be used throughout gestation; when dual therapy is required, clopidogrel (75 mg daily) is the preferred P2Y_12_ inhibitor [41]. Ticagrelor, prasugrel and intravenous GP IIb/IIIa antagonists have been reported only as single-case successes and should be reserved for situations where clopidogrel is unsuitable and maternal benefit clearly outweighs fetal risk [41]. In cancer patients, antiplatelet therapy is emerging as an adjunct to standard chemotherapy, immunotherapy and targeted agents, low-dose aspirin and P2Y_12_ antagonists have demonstrated the greatest clinical feasibility, reducing tumour-promoting platelet functions and enhancing drug penetration without causing systemic bleeding when used at cardioprotective doses [42]. Owing to the increased baseline risk of cancer-associated thrombosis and haemorrhage, any antiplatelet regimen must be individualised, avoiding prasugrel or vorapaxar (linked to excess cancer incidence) and integrating multidisciplinary monitoring, particularly in thrombocytopenic or perioperative settings [42].

Contemporary practice increasingly relies on standardized bleeding-risk scores to individualise antiplatelet duration and intensity. The five-item PRECISE-DAPT score (age, haemoglobin, white-blood-cell count, creatinine clearance, prior bleeding) accurately predicts BARC 3 or 5 bleeding up to 2 years after coronary stenting and is endorsed by ESC guidelines (class IIb) to identify patients in whom shorter (3–6 months) rather than prolonged DAPT may be preferable [43]. In the GLOBAL LEADERS and GLASSY cohorts (>14,000 patients), a PRECISE-DAPT ≥25 conferred a four-fold higher risk of major bleeding, with consistent performance whether patients received standard DAPT or ticagrelor monotherapy after 1 month [43]. The Academic Research Consortium High Bleeding Risk (ARC-HBR) definition combines 14 major and six minor clinical criteria—such as age ≥75 years, anaemia, prior intracranial haemorrhage, or severe chronic kidney disease—and is widely adopted in PCI trials to select patients who benefit from abbreviated or de-escalated antiplatelet strategies [43]. Both PRECISE-DAPT and ARC-HBR have been externally validated across diverse populations and antiplatelet regimens, and their integration into bedside decision-making has been shown to reduce bleeding without increasing ischaemic events.

## 5. Emerging Role of Pharmacist and Nurse Involvement in Antiplatelet Medication Management

Pharmacists play a critical role in optimizing antiplatelet therapy, contributing significantly to the clinical management of these drugs. This literature review explores the pharmacist’s role in managing antiplatelet therapy, particularly in monitoring medication regimens to identify potential drug interactions and bleeding risks. For instance, pharmacists can review patient medication profiles to detect interactions between antiplatelet drugs and other medications that may increase bleeding risk. A study indicated that platelet reactivity varies among patients, even with aspirin use [44]. Pharmacists can use this information to adjust drug therapy and minimize bleeding risks. They also provide valuable guidance on drug selection and dosing adjustments based on individual patient characteristics, such as age, weight, and renal function. According to von Pape *et al*. (2005) [45], adherence and dosage adjustments in long-term aspirin therapy influence platelet function, a factor pharmacists can leverage to optimize drug therapy. Pharmacists enhance patient adherence to antiplatelet therapy by educating patients on the importance of adherence, potential side effects, and the need for regular monitoring. Lee *et al*. (2005) [46] found that low-dose aspirin could increase aspirin resistance in patients with coronary artery disease. Pharmacists can use this insight to improve patient adherence through targeted education [46]. Additionally, pharmacists collaborate with other healthcare professionals to develop and implement protocols for bleeding risk assessment and management. They can work alongside physicians to establish guidelines for monitoring and managing bleeding complications in patients on antiplatelet therapy. A study by Healey *et al*. (2024) [47] emphasized the importance of such interdisciplinary collaboration in managing antiplatelet therapy for atrial fibrillation patients. Pharmacists contribute their expertise in these multidisciplinary teams. Furthermore, pharmacists play a key role in analyzing drug-drug interactions that may impact the efficacy and safety of antiplatelet therapy. For example, interactions between antiplatelet drugs and PPIs can affect platelet function. A systematic review and meta-analysis by Luo *et al*. (2023) [48] examined the efficacy and safety of concomitant PPI use with aspirin-clopidogrel DAPT in CHD, which pharmacists can use to assess and manage such interactions in clinical practice.

Pharmacists are integral to the clinical management of antiplatelet drugs through their expertise in drug therapy monitoring, patient education, and collaboration with the healthcare team. Their involvement in optimizing antiplatelet therapy and mitigating bleeding risks is vital for improving patient outcomes, and their skills are essential in enhancing the quality of care for patients receiving antiplatelet therapy.

Nurses are central to patient care, playing a vital role in monitoring for early signs of bleeding [49]. They routinely assess patients for symptoms such as unusual bruising, mucosal bleeding, and gastrointestinal signs indicative of hemorrhage. In patients with ACS receiving antiplatelet therapy, nurses’ diligent monitoring enables timely interventions, helping to prevent severe complications [50]. Nurses also collaborate with physicians to ensure the appropriate use of antiplatelet drugs [51], facilitating communication within the healthcare team to ensure prompt decision-making and interventions. In the AEGIS cluster randomized trial, Kurlander *et al*. [51] demonstrated that nurse-led interventions effectively resulted in the discontinuation of unnecessary antiplatelet medications or the initiation of PPIs in patients initially prescribed warfarin and antiplatelet therapy without gastroprotection. Nurse-led standardized anticoagulation follow-up promptly identified and corrected inappropriate combinations of antiplatelet drugs, significantly reducing patients’ bleeding risks [52]. Clinical practice guidelines highlight that nurses can significantly reduce potential bleeding events and ensure medication continuity by early identification of bleeding risks, prioritizing absorbable hemostatic materials, strengthening patient education, and maintaining 30-day follow-up records for antiplatelet drug-related epistaxis management [53]. These guidelines underscore the importance of multidisciplinary collaboration in optimizing antiplatelet therapy and improving patient outcomes. Zhang *et al*. [54] identified antiplatelet drug use as an independent risk factor for hemorrhage after intravenous thrombolysis, providing clinical nurses with a basis for evaluating hemorrhage risk in acute ischemic stroke patients post-thrombolysis. A longitudinal study found that a significant proportion of ACS patients were likely non-adherent to antiplatelet therapy within 30 days of discharge, offering valuable insights for nurses in designing targeted interventions to integrate adherence strategies into patients’ daily routines [55].

Nurses play an essential role in improving the quality of care for patients on antiplatelet therapy. However, as the literature indicates, there remain several areas that require more focused involvement of nurses. Future research should continue to explore and strengthen the roles of nurses in this field, ultimately improving the management of bleeding risks and patient care.

## 6. Discussion

**Fig. 3.**
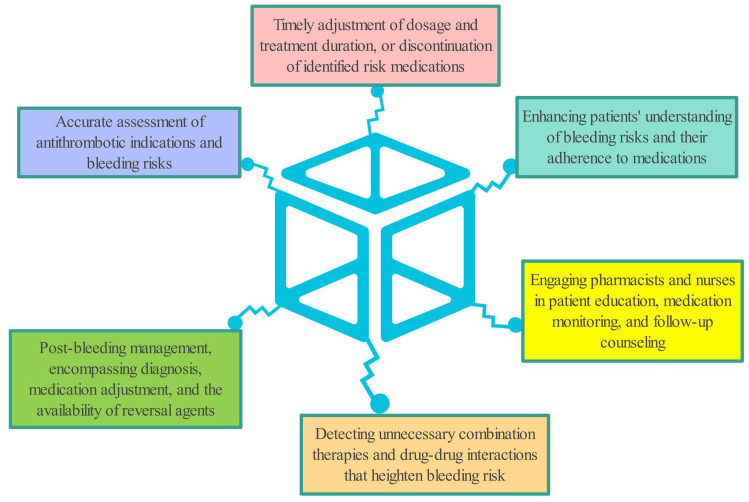
**Strategies to reduce bleeding risk or cope bleeding caused by antiplatelet drugs**.

Antiplatelet drugs are widely used to prevent and manage cardiovascular conditions and postoperative thrombosis, including coronary artery disease, acute ischemic stroke, and peripheral arterial disease, by inhibiting thrombus formation and improving clinical outcomes [56, 57, 58, 59]. However, these therapies increase the risk of bleeding, particularly in catastrophic events such as intracranial or gastrointestinal hemorrhages, which can significantly impact both survival and quality of life [60, 61]. Therefore, a thorough bleeding-risk assessment and proactive risk-mitigation strategies are essential when prescribing antiplatelet therapy (Fig. 3). Timely intervention—such as discontinuing the drug, administering blood transfusions, or performing surgery—may be necessary when bleeding occurs, ensuring that the benefits of antiplatelet therapy outweigh its risks. The radial artery approach can significantly reduce the incidence of PCI-related bleeding complications, shorten the length of hospital stay and lower the need for blood transfusion, especially for patients with a high risk of bleeding [62]. Additional procedural interventions likely ultrasound-guided vascular access has been shown to further reduce bleeding complications [63]. Clinical indications for antiplatelet therapy in key clinical trials or guidelines (Table 1).

**Table 1.** **Clinical indications for antiplatelet therapy in key clinical trials or guidelines**.

Indication	First-line regimen	Recommended duration	Evidence sources
Acute coronary syndrome—post-PCI	Aspirin + P2Y_12_ inhibitor (clopidogrel/ticagrelor)	12 months DAPT (shortened to 3–6 months if high bleeding risk)	ESC 2021 guidelines
Chronic coronary syndrome—post-PCI	Aspirin + clopidogrel	6 months DAPT (1–3 months if high bleeding risk)	ESC 2020/CCS-CAIC 2023 guidelines
Minor ischemic stroke or high-risk TIA	Aspirin + clopidogrel	3 months DAPT → single antiplatelet thereafter	AHA/ASA 2021 guideline
Transcatheter aortic valve replacement	Aspirin monotherapy	Lifelong	ESC 2021 guideline
Elective PCI with drug-eluting stent (low-bleeding-risk patients)	Aspirin + clopidogrel	1 month DAPT → clopidogrel monotherapy	STOPDAPT-2 trial
Secondary prevention after PCI (maintenance)	Clopidogrel monotherapy	Lifelong	HOST-EXAM trial

PCI, percutaneous coronary intervention; DAPT, dual antiplatelet therapy; ESC, European Society of Cardiology; CCS-CAIC, Canadian Cardiovascular Society-Canadian Association of Interventional Cardiology; TIA, transient ischemic attack; AHA/ASA, American Heart Association/American Stroke Association; STOPDAPT, Short and Optimal Duration of Dual AntiPlatelet Therapy; HOST-EXAM, Harmonizing Outcomes with Revascularization and Stents in Acute Myocardial Infarction - EXtended Antiplatelet Monotherapy.

This review provides the first comprehensive analysis of bleeding complications associated with antiplatelet agents, highlighting the critical roles of pharmacists and nurses in mitigating these risks. By synthesizing the most recent evidence from reputable databases, it offers a more contemporary and thorough perspective than previous studies. The review incorporates both observational studies and randomized controlled trials, significantly enhancing the depth and credibility of its findings. Additionally, it carefully examines key factors influencing bleeding risks, including drug type, dosage, treatment duration, and patient-specific variables such as age and comorbidities. Patient-related, drug-related, and procedural factors associated with increased bleeding risk (Table 2, Ref. [17, 23, 26, 62, 64, 65]). This nuanced analysis offers clinical patient management a more detailed understanding of how to mitigate bleeding risks in individual patients.

**Table 2.** **Patient-related, drug-related, and procedural factors associated with increased bleeding risk**.

Factor category	Specific factor	Reference(s)
Patient-related	Age ≥75 years	[26]
Patient-related	Anaemia (baseline Hb <120 g/L)	[26]
Patient-related	Type 2 diabetes	[26]
Patient-related	Frailty (per 1-grade increase)	[23]
Drug-related	Dual antiplatelet therapy vs aspirin alone	[64]
Drug-related	Antiplatelet therapy + OAC	[17]
Drug-related	Prolonged DAPT (>12 months)	[65]
Procedural	Femoral vs radial access for PCI	[62]

OAC, oral anticoagulant.

Antiplatelet agents primarily aim to prevent vascular reocclusion, yet their safety and efficacy can vary greatly due to individual differences [66]. Sex-specific disparities have been noted in platelet reactivity, patient management strategies, and clinical outcomes following treatment with aspirin, P2Y_12_ inhibitors, or DAPT [67]. Key factors influencing the use of antiplatelet drugs include the type of ACS, time to angiography, drug onset of action, patient clinical profile (e.g., surgery or oral anticoagulation), bleeding risk, and tolerance to DAPT [68]. Recent studies have explored aspirin-free approaches for secondary prevention. A recent meta-analysis comparing P2Y_12_ inhibitor monotherapy with prolonged (≥12 months) DAPT (including aspirin) after PCI found that the P2Y_12_ monotherapy group had a lower risk of major bleeding and no increased risk of stent thrombosis, all-cause mortality, or stroke [69]. Systematic reviews and meta-analyses have indicated that the combination of clopidogrel and aspirin offers greater efficacy but comes with a higher bleeding risk (risk ratio: 2.21, 95% confidence interval: 1.48–3.32) compared to monotherapy [64]. Prolonged combined antiplatelet therapy (beyond 1 month) was also associated with a higher risk of hemorrhagic stroke [65]. A recent review demonstrates that race and geography significantly modulate response to antiplatelet therapy [70]. East Asians carry a three-fold higher prevalence of cytochrome P450 2C19 (CYP2C19)*2 loss-of-function alleles than whites, leading to markedly reduced clopidogrel efficacy and a lower approved prasugrel dose (3.75 mg) in Japan. Blacks exhibit higher baseline platelet reactivity and inflammation, coupled with lower aspirin utilization, predisposing to worse outcomes despite standard dosing. Conversely, ticagrelor shows consistent efficacy across regions, although East Asians achieve higher drug levels without excess bleeding. A 2023 expert consensus states that after risk stratification, low-ischaemia/high-bleeding-risk patients can safely abbreviate DAPT: stop aspirin 1–3 months post-PCI and continue P2Y_12_ inhibitor monotherapy, or switch to aspirin alone at 3–6 months [71]. This approach is best documented in East Asians, the elderly (≥75 years), and those with moderate-to-severe chronic kidney disease (CKD), and can be further guided by platelet-function or genotype-directed de-escalation [71]. Therefore, antiplatelet therapy regimens should be tailored to individual patients based on disease type, onset timing, race, genotype, and susceptibility to hemorrhagic complications. No specific reversal agent exists for patients on antiplatelet therapy who develop spontaneous or traumatic intracranial hemorrhage [72]. In emergency settings, platelet transfusion (1 pool, >3 × 10^9^ platelets/L) combined with desmopressin (0.3 µg/kg) can quickly restore platelet function. Typically, up to 5 units of platelets are required for adequate clot formation, with desmopressin potentially repeated every 12 hours up to a maximum of six doses [72].

Pharmacists and nurses collaborate to significantly enhance patient outcomes through their distinct roles [73, 74]. Pharmacists focus on medication therapy management, drug interaction analysis, patient education, and protocol development, while nurses excel in patient monitoring, clinical decision support, patient education, and bleeding risk assessment [75]. This review enriches the discourse on the roles of multidisciplinary teams in hemorrhage risk management by providing more concrete evidence of their contributions. Unlike earlier studies, which acknowledged the importance of pharmacists and nurses in drug therapy [75], this review highlights the specialized expertise of pharmacists in drug interaction analysis and nurses’ acute vigilance in detecting early signs of bleeding. These advances emphasize the tangible impact of their roles in improving patient outcomes.

However, this review has limitations. The search strategy may have excluded some less commonly used antiplatelet agents or bleeding-related terms. As a narrative review, it lacks a systematic evaluation process (e.g., Preferred Reporting Items for Systematic Reviews and Meta-Analyses (PRISMA) flowchart, two-person independent screening, and conflict resolution), making it difficult to assess the risk of selection bias. The studies reviewed were conducted in various countries, yet there is limited discussion on dose-exposure relationships, ethnic disparities, and related factors. Additionally, there is a scarcity of research on special populations, such as individuals with impaired liver or kidney function, tumors, or those who are pregnant. Currently, the research lacks large-sample, randomized controlled trials, making it impossible to quantify the cost-effectiveness, resource allocation, and long-term outcomes of interventions by pharmacists or nurses. Moreover, the review predominantly focuses on the roles of pharmacists and nurses within hospital settings, highlighting the need for further research to explore their roles in community or primary care environments.

Future research should focus on developing more precise bleeding risk assessment tools tailored to different patient populations and antiplatelet regimens. By employing dual reviewer screening and independent quality assessments, a rigorous systematic review with clearly defined inclusion criteria could minimize bias. Larger prospective studies are essential to confirm the effectiveness of multidisciplinary interventions in reducing bleeding complications and improving patient outcomes. Additionally, investigating the cost-effectiveness of these interventions would provide valuable insights for healthcare policymakers. The integration of advanced technologies, such as artificial intelligence for real-time bleeding risk monitoring, presents a promising direction for future research. Timely interventions, such as drug withdrawal, blood transfusion, or surgery, may be necessary when bleeding events occur to balance the benefits and risks of antiplatelet therapy. Therefore, careful bleeding-risk assessment and proactive risk-mitigation strategies are crucial when prescribing antiplatelet therapy. Pharmacists and nurses, as essential members of the healthcare team, play pivotal roles in optimizing patient outcomes. Future research should focus on optimizing the timing of antiplatelet drug initiation based on individual patient characteristics. For patients with high bleeding risk or complex comorbidities, dynamic imaging assessments and real-time monitoring could be employed to evaluate vascular reperfusion and bleeding risk, enabling precise timing for initiating antiplatelet therapy. Furthermore, additional refinement is needed in the combination and dosage control of antiplatelet drugs when used with other antithrombotic agents to balance efficacy and bleeding risk. Furthermore, mounting evidence indicates that CYP2C19 polymorphisms, platelet-function-test results, and clinical factors such as age and renal function jointly determine individual responsiveness to P2Y_12_ inhibitors. Integrating genotyping with bleeding-risk scores therefore allows dynamic tailoring of agent selection, initial dosing, and DAPT duration (1–3 vs 12 months) [71]. It suggests that this “genotype-phenotype-clinical” triad can substantially reduce major bleeding without increasing thrombotic events, and warrants prospective validation. Addressing these gaps through systematic reviews, large-scale randomized controlled trials, and real-world evidence studies will be critical for advancing clinical practice.

## 7. Conclusion

This review enhances our understanding of the bleeding risks associated with antiplatelet therapies and emphasizes the importance of pharmacist and nurse involvement in managing these risks. Future research should build on these findings to further refine antiplatelet therapy strategies and risk mitigation approaches.

## Abbreviations

ACS, acute coronary syndrome; ADP, adenosine diphosphate; ASCVD, atherosclerotic cardiovascular disease; ATP, adenosine triphosphate; cAMP, cyclic adenosine-3’,5’-monophosphate; CHD, coronary heart disease; DAPT, dual antiplatelet therapy; GP, glycoprotein; MACE, major adverse cardiovascular events; NSAIDs, non-steroidal anti-inflammatory drugs; PCI, percutaneous coronary intervention; PPI, proton pump inhibitors; TXA_2_, thromboxane A2.
